# Single cell lineage reconstruction using distance-based algorithms and the R package, DCLEAR

**DOI:** 10.1186/s12859-022-04633-x

**Published:** 2022-03-24

**Authors:** Wuming Gong, Hyunwoo J. Kim, Daniel J. Garry, Il-Youp Kwak

**Affiliations:** 1 Lillehei Heart Institute, University of Minnesota, Minneapolis, USA; 2 Department of Computer Science and Engineering, Korea University, Seoul, Republic of Korea; 3 Department of Applied Statistics, Chung-Ang University, Seoul, Republic of Korea

**Keywords:** Cell lineage tracing, Lineage reconstruction, Machine learning, Simulation

## Abstract

**Background:**

DCLEAR is an R package used for single cell lineage reconstruction. The advances of CRISPR-based gene editing technologies have enabled the prediction of cell lineage trees based on observed edited barcodes from each cell. However, the performance of existing reconstruction methods of cell lineage trees was not accessed until recently. In response to this problem, the Allen Institute hosted the Cell Lineage Reconstruction Dream Challenge in 2020 to crowdsource relevant knowledge from across the world. Our team won sub-challenges 2 and 3 in the challenge competition.

**Results:**

The DCLEAR package contained the R codes, which was submitted in response to sub-challenges 2 and 3. Our method consists of two steps: (1) distance matrix estimation and (2) the tree reconstruction from the distance matrix. We proposed two novel methods for distance matrix estimation as outlined in the DCLEAR package. Using our method, we find that two of the more sophisticated distance methods display a substantially improved level of performance compared to the traditional Hamming distance method. DCLEAR is open source and freely available from R CRAN and from under the GNU General Public License, version 3.

**Conclusions:**

DCLEAR is a powerful resource for single cell lineage reconstruction.

## Background

A multicellular organism or animal develops from one to two to four to an exponential number of cells. One of the challenges is the ability to predict the lineage tree representing the cell differentiation process starting from the single parental cell, based on cells extracted from the adult body. McKenna et al. [1] used gene editing technology and the immune system (CRISPR-CAS9) as the basis for proposing a methodology called GESTALT for estimating a cell-level lineage tree using the data generated using CRISPR-CAS9 barcode edits from each cell. Subsequently, Raj et al. [2] proposed scGESTALT, which further considers cell type identification as an extension of GESTALT. Researchers have developed a number of additional CRISPR recorder-based technologies [3–5].

This field lacked experimentation or analyses regarding the effectiveness of these proposed algorithms for comprehensive evaluation. To improve the evaluation process, the Allen Institute established The Cell Lineage Reconstruction DREAM Challenge [6]. The Allen Institute proposed three different sub-challenges to benchmark reconstruction algorithms of cell lineage trees: (1) the reconstruction of in vitro cell lineages of 76 trees with fewer than 100 cells; (2) the reconstruction of an in silico cell lineage tree of 1000 cells; (3) the reconstruction of an in silico cell lineage tree of 10,000 cells. Our proposed *DCLEAR* method won sub-challenges 2 and 3 of this challenge competition.

We outline and define the problem setting addressed in cell lineage reconstruction in the next section. We then present two core methods for distance matrix construction and outline how *DCLEAR* software may be applied to a simulated dataset.

## Implementation

### Problem setup

**Fig. 1:**
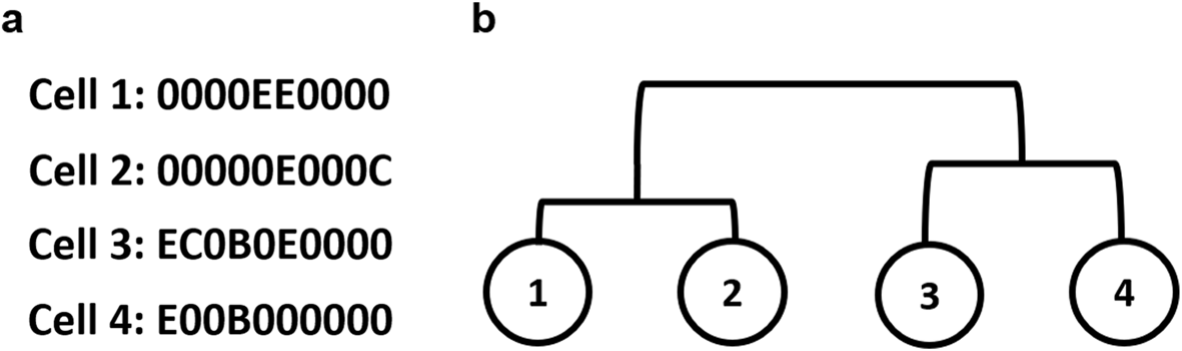
**a** is the observed set of cell sequences, and **b** is the cell lineage tree. The goal is to predict the cell lineage tree in (**b**) using the cell sequences in (**a**)

Assume we have *n* number of training data pairs. Each data pair consists of a set of cell sequences and a true cell lineage tree. One example of such a pair is illustrated in Fig. 1.

For the *i*th data pair, let $m_i$ be the number of cell sequences in the *i*th data pair and let *t* be the sequence length. Let the sequence information in data pair *i* be written as $C^i$, an $m_i\times t$ matrix. The matrix element $C^i_{jk}$ describes the *j*th sequence and the *k*th letter of the *i*th training data pair. Furthermore, we represent $L_i$, the cell lineage tree structure, in the form of a Newick format string.

For example, if Fig. 1 represents *i*th data pair, (a) shows the sequence information. It has $m_i=4$ cell sequences, each sequence length has a length (*t*) of 10, and the first letter of the 3rd sequence is $C^i_{3,1}=\text {E}$. The cell lineage tree is shown in Fig. 1b. In Newick format, $L_i = ((1:0.5,2:0.5):2,(3:1.2,4:1):2)$.

We built our model ($m(C;{\hat{\theta }})$) with *n* training pairs. The input matrix, *C* is an $m_i\times t$ sequence information matrix. The output is the Newick format string representing the tree structure while $\theta$ represents the parameter set related to model $m(C;\theta )$, and ${\hat{\theta }}$ represents the estimated parameter with *n* training data pairs.

To evaluate the model, we have *l* number of unused data. The loss is indicated as $L(m(C^i;{\hat{\theta }}), L_i)$. The loss increases when the predicted tree structure ($m(C;{\hat{\theta }})$) differs from the true tree structure ($L_i$). We use the averaged loss metric (*AL*) defined below to evaluate the model $m(C;{\hat{\theta }})$.

1 $$\begin{aligned} AL = \frac{\sum _{i=1}^{l} L(m(C^i;{\hat{\theta }}), L_i)}{l}. \end{aligned}$$

Next, we need to define the quantity $L(L_1, L_2)$ that represents the dissimilarity between the two lineage trees, $L_1$ and $L_2$. The two widely used measures of this dissimilarity are the Robinson–Foulds (RF) distance [7] and the Triplet distance [8].

**Fig. 2:**
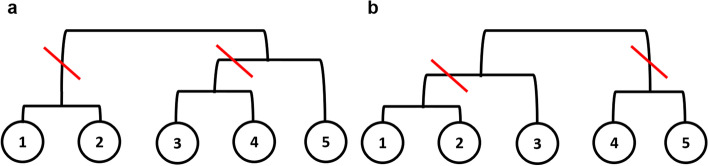
Two different trees, **a** $L_1$ and **b** $L_2$, are presented to explain how the RF and triplet distances are defined. There are two possible cuts for each tree to separate items in the tree with two different sets of more than two items. The red slanted lines represent the possible cuts for separation

Figure 2 represents two lineage trees, $L_1$ and $L_2$. The possible separations for tree 1 are $\{1,2\}, \{3,4,5\}$ and $\{1,2,5\}, \{3,4\}$, and the possible separations for tree 2 are $\{1,2\}, \{3,4,5\}$ and $\{1,2,3\}, \{4,5\}$. Among the four possible separations, $\{1,2\}, \{3,4,5\}$ in tree 1 and tree 2 are concordant separations. The RF distance is defined as the total number of concordant separations divided by the total number of separations. As an example: the RF score for Figure 2 is 2/4 = 0.5.

For triplet distance calculations, we sample three items among all items in the tree. We determine $_5C_3=10$ possible cases. We check whether the tree structure of the three items in tree 1 and tree 2 are the same. For example, the five cases of $\{1,2,3\}, \{1,2,4\}, \{1,2,5\}, \{1,4,5\}$ and $\{2,4,5\}$ have the same tree structure in both tree 1 and tree 2. The triplet score is defined as the number of cases with the same tree structure divided by the number of possible cases. Accordingly, the triplet score for our example is 5/10 = 0.5.

We will use $AL_{RF}$ to denote the RF distance and $AL_{TP}$ to denote the triplet distance.

### Overview of the modeling architecture

**Fig. 3:**
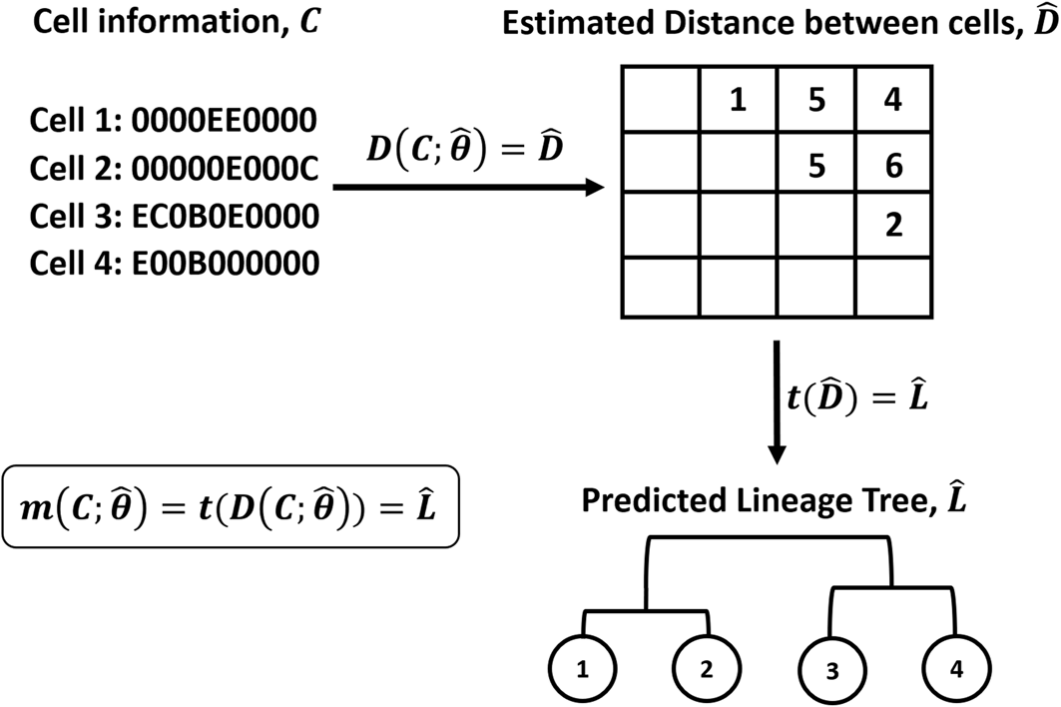
Overview of our modeling architecture. Our model function $m(C;\theta )$ is divided into two parts: (1) estimating the distance between cells and (2) constructing a tree using the distance matrix

The two main issues that need to be answered in the lineage reconstruction problem are (1) how should the model $m(C;\theta )$ be built and (2) how should ${\hat{\theta }}$ be estimated? Our modeling architecture for $m(C;\theta )$ is described in Fig. 3. We divide the model into two parts: (1) estimating the distance between cells and (2) constructing a tree using a distance matrix. Let *D*(*C*) be a function for estimating the distance matrix for an $m \times t$ input sequence matrix, *C*, and let *t*(*D*) be a function for predicting the lineage tree for an $m \times m$ distance matrix, *D*. Note that a knowledge of the triangular components in *D* is sufficient for defining the distance matrix. Subsequently, the challenge becomes how $D(\cdot )$ and $t(\cdot )$ should be defined.

### Choice of distance

One notion for calculating the distance is to define the distance function for the two sequences. Let $d(C_{i\cdot }, C_{j\cdot }; \theta )=d_{ij}$ be the calculated distance between the *i*th cell and the *j*th cell obtained from the given cell information matrix *C*. The quantities $C_{i\cdot }, i = 1,\cdots ,m$ represent the *i*th cell vector taken from *C*. The quantity $d_{ij}$ is the (*i*, *j*)th element of the cell distance matrix *D*. The next challenge becomes how $d(\cdot , \cdot )$ should be defined.

#### Hamming distance method

One naive approach to modeling $d(\cdot , \cdot )$ is to compute the Hamming distance expressed in equation (2):

2 $$\begin{aligned} d_H(C_{i\cdot }, C_{j\cdot }) = \sum _{l=1}^{t} 1(C_{il}\ne C_{jl}), \end{aligned}$$

where $1{(\text {condition})}$ is an indicator function with a value of 1 if the given condition is true and 0 otherwise. Note that the Hamming distance $d_H(C_{i\cdot }, C_{j\cdot })$ simply counts unit differences between the two sequences $C_{i\cdot }$ and $C_{j\cdot }$.

**Fig. 4:**
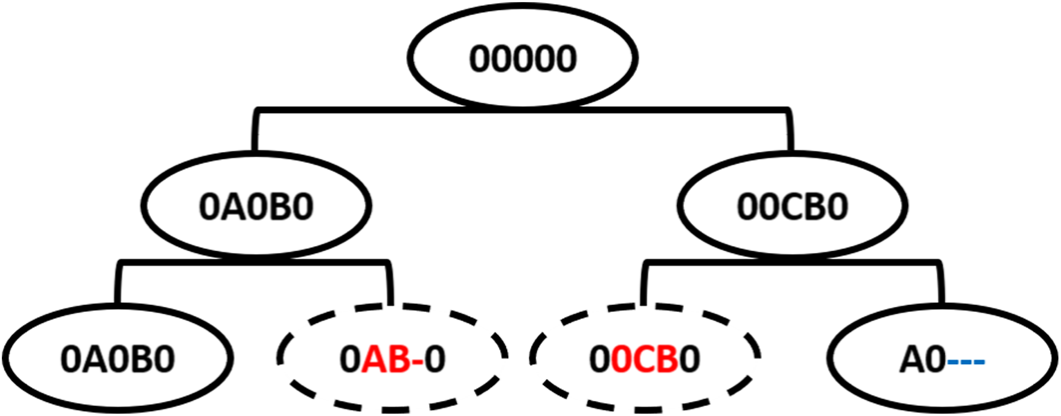
Example of cell differentiation process

The simple calculation of the Hamming distance does not meet the challenges of the present study. Consider the cell differentiation process illustrated in Fig. 4. Let the 2nd and the 3rd leaf cells (dotted) have $C_{2\cdot } = \text {0AB-0}$ and $C_{3\cdot }= \text {00CB0}$. The corresponding Hamming distance is $d_H(C_{2\cdot }, C_{3\cdot }) = 3$. However, it is not reasonable to assign equal weights to each target position. This deficiency is addressed by the weighted Hamming approach described in the following sub-sub-section.

The code for using the Hamming distance method is available from the *phangorn* package [9] using the dist.hamming function.

#### Weighted Hamming distance (WHD) method

An example of the cell diffusion process is illustrated in Fig. 4. Consider the objective of reconstructing the lineage tree based on information about the leaf nodes. What would be an appropriate measure for calculating the distance between 0AB-0 and 00CB0 (see the dotted circles in Fig. 4)? The character difference between ‘A’ and ‘0’ would be less than the character difference between ‘B’ and ‘C’ as the initial state ‘0’ can be differentiated to ‘A’ or to any other outcome states, whereas states ‘B’ and ‘C’ cannot be differentiated to other outcome states. In addition, the missing state ’-’ maybe any other state. Seeking a mathematical formula to accommodate these nuances, we propose the weighted Hamming distance (WHD) method:

3 $$\begin{aligned} d_{WH1}(C_{i\cdot }, C_{j\cdot }) = \sum _{l=1}^{t} w_{C_{il}}w_{C_{jl}}1(C_{il}\ne C_{jl}), \end{aligned}$$

where, $C_{il}$ is the *l*th character in the *i*th cell sequence, and $w_{C_{il}}$ is a weight associated with the character $C_{il}$. The code for calculating the WHD method is available as the dist_weighted_hamming function in the *DCLEAR* package. For the estimation of weight parameters, we used Bayesian hyperparameter optimization using the BayesianOptimization function in the *rBayesianOptimization* package [10].

#### k-mer replacement distance (KRD) method

The algorithm used to compute the k-mer replacement distance (KRD) method first uses the prominence of mutations in the character arrays to estimate the summary statistics used for the generation of the tree to be reconstructed. These statistics include the mutation rate, the mutation probability for each character in the array, the number of targets, and the number of cells. These estimated parameters were combined with pre-defined parameters, such as the number of cell divisions, to simulate multiple lineage trees starting from the non-mutated root. To generate trees with sizes and mutation distributions similar to the target tree, we generated 1000 lineage trees, each with 16 cell divisions of 216 leaves, a mutation rate of 0.1, arrays of 200 characters, 200 cells, and 30 states (‘A’-‘Z’ to ‘a’-‘c’, with an outcome probability following a Gamma distribution with a shape of 0.1 and a rate of 2). Different possibilities for the k-mer distance method were then estimated from the simulated lineage trees and used to compute the distances between the input sequences in the character arrays of internal nodes and tips. The KRD method is available from the *DCLEAR* package using the dist_replacement function.

### Tree construction

We use existing methods such as Neighbor-Joining (NJ), UPGMA, and FastMe [11–13] for tree construction from the estimated distance matrix, *D*. The NJ method is implemented as the nj function in the Analysis of Phylogenetics and Evolution (*ape*) package, UPGMA is implemented as the upgma function in the *phangorn* package, and FastMe is implemented as fastme.bal, and fastme.ols in the *ape* package.

## Results

This section reports the experimental results of applying the Hamming distance, WHD, and KRD methods using existing tree construction methods (NJ, UPGMA, and FastMe). For the use of NJ, UPGMA, and FastMe, the nj function in the *ape* package [14] was used for the NJ method, the upgma function in the *phangorn* package [9] was used for the UPGMA method, the fastme.ols function in the *ape* was used for the FastMe method, and the fastme.bal function in the *ape* was used for FastMe with tree rearrangement.

### Datasets

#### Simulation dataset

The simulation dataset was generated from our simulation code. Parameter settings for the simulation were as follows:

1. Number of targets: 50, 100, and 200
2. Mutation probability: 0.02, 0.04, 0.06, 0.08, and 0.1
3. Number of cells: 100
4. Dropout probability: 0, 0.05, and 0.1
5. Number of cell division: 16
6. Existance of point deletion: True, False

We iterated all 90 combinations ($3 \times 5 \times 3 \times 2$) of the parameter 5 times, result in 450 simulation datasets. For each iteration, We generated five training sets and one evaluation set. The averaged performance of the 450 evaluation sets was reported.

#### Sub-challenge 2 and 3 datasets

The sub-challenge 2 and 3 datasets can be downloaded from the competition website of the Allen Institute Cell Lineage Reconstruction Challenge at https://www.synapse.org/#!Synapse:syn20692755 (accessed on 17 September 2021). The sub-challenge 2 dataset (the dataset for C.elegans cells) contained a 1000 cell tree from the 200 mutated/non-mutated targets in each cell induced by simulation, and the sub-challenge 3 dataset (the dataset for mouse cells) had a 10,000 cell tree from the 1000 mutated/non-mutated targets in each cell induced by simulation. The different simulation models were used for sub-challenges 2 and 3. Both datasets had 100 trees. We randomly divided the sub-challenge dataset into 75 trees for training and 25 trees for evaluation.

#### Experimental results

**Fig. 5:**
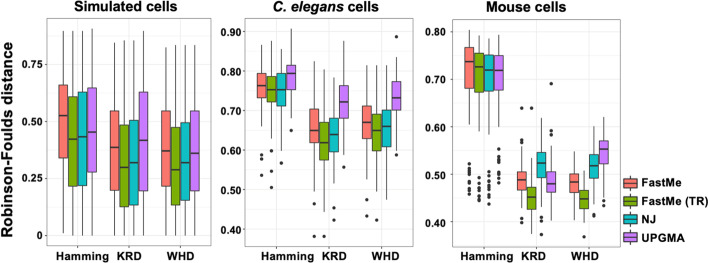
Experimental result

We outlined our experimental results in Fig. 5. The y-axis represented the RF distance, and the x-axis accommodated the different models. The Hamming distance, the KRD, and the WHD methods were used for distance calculation. NJ, UPGMA, and FastMe were methods for tree construction. For all three datasets, the KRD and the WHD methods displayed improved performance compared to the Hamming distance method. The performance achieved with the KRD was similar to that achieved with WHD. Among the tree construction methods, FastMe with tree rearrangement displayed improved performance compared to the other tree construction methods.

## Discussion

In these experiments, we only compared the Hamming distance, the WHD, and the KRD methods using the three datasets. Gong et al. [6] presented the comparison of the WHD and the KRD with the other methods that participated in the Cell Lineage Reconstruction Dream Challenge (2020). Our proposed WHD method was used for sub-challenge 3, and the KRD method was used for sub-challenge 2. DCLEAR won both sub-challenges 2 and 3 but did not participate in sub-challenge 1. The sub-challenge 1 dataset has only ten target positions with two outcome states. The WHD and KRD methods were more powerful techniques with more complex settings, such as the existence of a dropout interval, the existence of missing target positions, and a larger number of outcome states. With the small target positions and the small outcome states of sub-challenge 1, the gain achieved by using the WHD and the KRD methods was not high. We were not able to show a comparison for sub-challenge 1 as DCLEAR did not participate in that sub-challenge. Thus, the future work of DCLEAR will contain an extension of the WHD and the KRD methods with small target and small outcome settings. Furthermore, for the WHD method, the hyperparameter tuning was performed using BayesianOptimization because the loss was not differentiable with respect to weight parameters. We could utilize the surrogate loss to address this non-differentiable loss [15].

## Conclusions

*DCLEAR* is a powerful resource for single cell lineage reconstruction.

## Figures and Tables

**Fig. 6:**
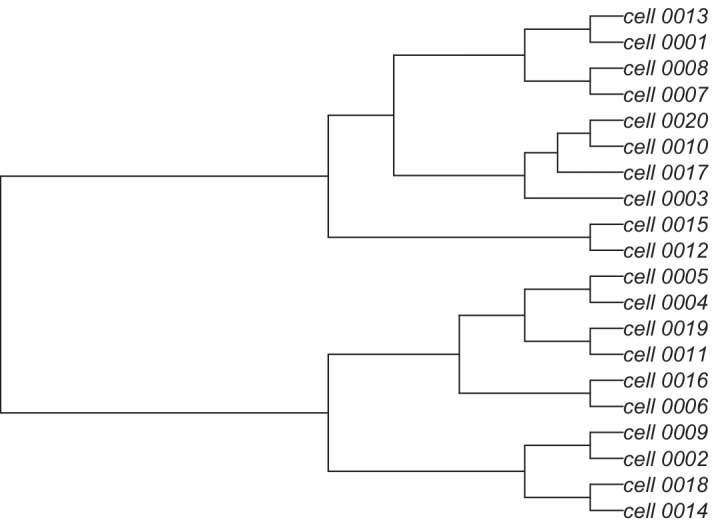
Tree structure of 20 leaf cells

**Fig. 7:**
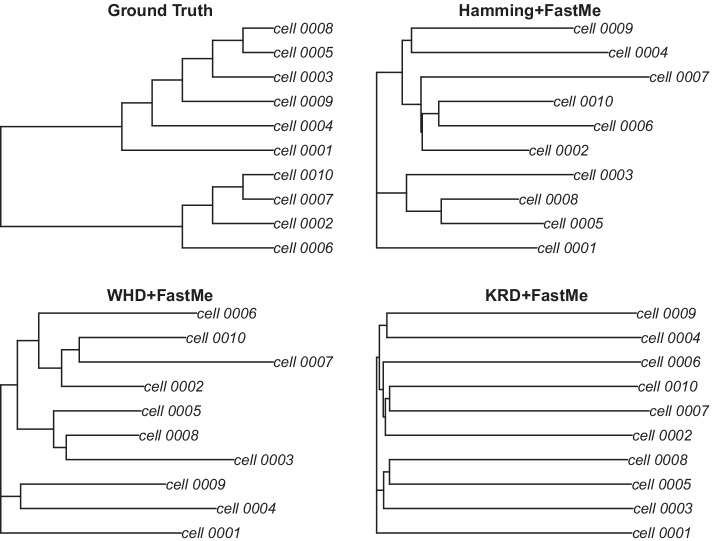
Tree structure of 10 leaf cells, as determined for various standard distance measures

## Data Availability

DCLEAR is available from R cran at https://cran.r-project.org/web/packages/DCLEAR/index.html, and Github at https://github.com/ikwak2/DCLEAR Datasets are downlaodable from the challenge website, https://www.synapse.org/#!Synapse:syn20692755/wiki/. after agreeing the terms and conditions. The R/DCLEAR package is free software; you can redistribute it and/or modify it under the terms of the GNU General Public License, version 3.

## Appendix

### Simulating trees with barcodes

For a simple illustration of lineage reconstruction, we simulated data using the sim_seqdata function in the *DCLEAR* package coded using the *R* [16] language. The sim_n parameter represented the number of cell samples. The prob_state parameter represented the cell state probability. The parameter m represented the number of targets. The parameter n_s represented the number of outcome states which equals the length quantified by prob_state. The parameter mu_d represented the mutation probability for each target position on every cell division. The parameter d represented the number of cell divisions. Finally, the parameter p_d represented the dropout probability of each target position for every cell division. The code is outlined below:

With this code, we generate a lineage tree of 20 leaf barcodes with 10 target positions. The sD$seqs contains the sequence information, and the sD$tree has the true tree structure:

We can also print character information of the simulated barcodes.

The initial cell state is ‘0000000000’. There are $$d=12$$ number of cell divisions resulting in $$2^{12}$$ leaf nodes. Every target position changes to a different outcome state for each cell division with a probability $$mu\_d = 0.03$$. The mutational positions randomly change to different outcomes, which follows a multinomial distribution with a probability $$p=out\_prob$$. Every target position of leaf nodes randomly changes to a missing state (’-’) with a probability $$p_d = 0.005$$. Finally, $$simn = 20$$ cells are randomly sampled. The true lineage tree structure of 20 cells ($$simn = 20$$) is recorded in sD$tree. The tree structure corresponding to $$simn = 20$$ cells is illustrated in Fig. 6.

Subsequently, we prepared five lineage trees as a training dataset. Each lineage tree has 10 leaves with 40 barcode target positions:

SDs is a list of 5 lineage trees. Each item of the SDs list is generated using sim_seqdata function:

The ten barcodes of the first training data are shown below.

We observed interval missing (dropout) events by marking them with a sequence of hyphens. Weight parameters for the WHD method were trained using the WH_train function. We specified the weight for the initial state and the weight for the dropout state.

We printed out the state characters as indicated below:

Since the initial state (‘0’) is in the first position and the dropout state (‘-’) is in the second position, we specify loc0=1 and locDroputout = 2:

We simulated one evaluation datum and compared the ground truth tree with three generated trees using the Hamming, WHD, and KRD distances with the FastMe algorithm for tree construction:

First, we calculated the distance matrix using the Hamming distance, the WHD, and the KRD methods:

Second, we constructed the tree using the FastMe algorithm with the tree rearrangement using fastme.bal function. The ground truth tree and the three generated trees were demonstrated in Fig. 7. As an alternative to fastme.bal function, we could use different tree construction algorithms using nj, upgma, and fastme.ols functions.

As outlined in Fig. 7, it is challenging to compare and determine which generation is the optimal one. One useful metric for making a determination is the RF distance. We calculated and compared the RF distances for the various generations. The RF distances were calculated and compared as indicated below:

The lower the value of the RF distance between the ground truth tree and the generated tree, the greater the similarity between the ground truth tree and the generated tree. Thus, for the given example data, the KRD method produced optimal results.

## Abbreviations

DCLEAR: Distance based Cell LinEAge Reconstruction
CRISPR: Clustered regularly interspaced short palindromic repeats
R: Statistical Language R
CRAN: Comprehensive R archive network
GESTALT: Genome editing of synthetic target arrays for lineage tracing
scGESTALT: An extended version of GESTALT considering single-cell RNA sequencing data
AL: Averaged loss metric
RF: Robinson–Foulds distance
WHD: Weighted Hamming distance
KRD: k-mer replacement distance
NJ: Neighbor-joining
UPGMA: Unweighted pair group method with arithmetic mean
FastMe: Fast distance-based phylogeny inference program
